# Initial characterization of M_2_-muscarinic receptor overexpressing mouse heart

**DOI:** 10.1007/s00210-025-04502-3

**Published:** 2025-08-13

**Authors:** Ulrich Gergs, Igor B. Buchwalow, Britt Hofmann, Jaromir Myslivecek, Katerina Janisova, Katarina Hadova, Franziska Schettler, Max Keller, Jan Klimas, Joachim Neumann

**Affiliations:** 1 Institute for Pharmacology and Toxicology, Medical Faculty, Martin-Luther-University Halle-Wittenberg (UG, JN), Magdeburger Straße 4, 06097 Halle (Saale), Germany; 2 Institute for Hematopathology (IBB), Fangdieckstraße 75a, D-22547 Hamburg, Germany; 3 Scientific and Educational Resource Center for Molecular Morphology, Peoples’ Friendship, University of Russia (IBB), Miklukho-Maklaya Street 6, RU-117198 Moscow, Russia; 4 Institute of Physiology, Charles University (JM, KJ), Albertov 5, CZ-128 00 Prague 2, Czech Republic; 5 Department of Pharmacology and Toxicology, Faculty of Pharmacy, Comenius University (KH, JK), Odbojárov 10, SK-83232 Bratislava 3, Slovak Republic; 6 Institute of Pharmacy, University of Regensburg (MK, FS), Universitätsstraße 31, D-93053 Regensburg, Germany; 7 Department of Cardiac Surgery, Medical Faculty, Martin-Luther-University Halle-Wittenberg (BH), Ernst-Grube-Straße 40, D-06097 Halle (Saale), Germany

**Keywords:** Human M_2_-muscarinic receptors, Arrhythmia

## Abstract

There are five muscarinic receptor subtypes expressed in the human heart. The main subtype is the M_2_-muscarinic receptor. We hypothesized that overexpression of the M_2_-muscarinic receptor should augment any contractile effects that are M_2_-muscarinic receptor–mediated. Therefore, we generated a transgenic mouse with overexpression of the human M_2_-muscarinic receptor under the control of the heart-specific α-myosin heavy chain promoter (M_2_-TG). We performed contraction experiments with electrically stimulated (1 Hz) left atrial preparations (LA) and spontaneously beating right atrial preparations (RA) from adult M_2_-TG or from adult wild-type littermate mice (WT). We confirmed the expression of the human M_2_-muscarinic receptor in the mouse heart by reverse transcription polymerase chain reaction (RT-PCR) and radioligand binding experiments at cardiac membranes and tissue sections. We did not detect differences in hematoxylin/eosin staining or Masson/Goldner staining between M_2_-TG and WT. We noticed that carbachol (10 nM–10 µM cumulatively applied) alone or in the presence of 1 µM isoprenaline reduced the force of contraction (FOC) to a similar extent in LA from M_2_-TG and WT. The beating rate in RA was similarly decreased by carbachol alone or by carbachol in the presence of 1 µM isoprenaline in M_2_-TG and WT. Overall, the number of RA that displayed absolute arrhythmias was higher in atria from M_2_-TG compared to atria from WT. No arrhythmias were noted in LA from M_2_-TG or WT. Stimulation of human M_2_-muscarinic receptors induced absolute atrial arrhythmias more often in RA from M_2_-TG than in RA from WT. Overexpressed M_2_-muscarinic receptors were silent to the force and beating rate.

**Supplementary Information:**

The online version contains supplementary material available at 10.1007/s00210-025-04502-3.

## Introduction

In the human heart, the M_2_-muscarinic receptors are the most highly expressed variant of the muscarinic acetylcholine receptor family. Yet, four other muscarinic receptors are detected in the atrium and ventricle of the human heart (Dhein et al. 2001). M-cholinoceptor agonists per se lead to negative inotropic (NIE) and negative chronotropic effects (NCE). After β-adrenergic stimulation, these effects are more accentuated and were therefore also studied here. The NIE and NCE are blocked by M_2_-muscarinic receptor antagonists (Du et al.^1994^; Dhein et al. 2001). Likewise, in mice with general deletion of M_2_-muscarinic receptors, the NCE and NIE of carbachol or acetylcholine were abolished (Gomeza et al. 1999, Stengel et al. 2000). Increased expression of cardiac M_2_-muscarinic receptors in binding studies has been reported in children who died from the Sudden Infant Death Syndrome (SIDS: Livolsi et al. 2010a, 2010b). In human ageing hearts, the density of M_2_-muscarinic receptors declines, whereas elevated M_2_-muscarinic receptor expression levels were found in patients with idiopathic cardiomyopathy (Brodde et al. 1998; Le Guludec et al. 1997). Hence, decreased and increased cardiac expression of M_2_-muscarinic receptors occurs in humans. M_2_-muscarinic receptors are of potential clinical relevance because they can induce deadly arrhythmias (Shi et al. 2004; Livolsi et al. 2010b). Interestingly, the expression of M_2_-muscarinic receptor was higher in the heart of ageing rabbits and correlated with an increased incidence of cardiac arrhythmias (Yang et al. 2009). Further evidence for a possible role of M_2_-muscarinic receptor in the genesis of cardiac arrhythmias comes from studies in dogs: right atria were paced at 400 beats per minute for 1 week. This led to a reduction in the expression of M_2_-muscarinic receptors in left atria demonstrated by Western blotting. Thus, arrhythmias (here tachycardia) can decrease the expression of M_2_-muscarinic receptors in the mammalian atrium (Yeh et al. 2007). However, these data do not prove a causal relationship between chronic arrhythmias and reduced expression of M_2_-muscarinic receptors because the authors reported that besides M_2_-muscarinic receptors also M_3_- and M_4_-muscarinic receptors were downregulated in the left atria of the tachypaced dogs. They speculated M_3_- and M_4_-muscarinic receptors may be more relevant than M_2_-muscarinic receptors for the genesis of arrhythmias in their canine system (Yeh et al. 2007). Thus, it remained uncertain what role the overexpressed M_2_-muscarinic receptor plays in the genesis of arrhythmias or whether overexpressed M_2_-muscarinic receptor is not directly involved in cardiac remodeling caused by long lasting arrhythmias (Yeh et al. 2007).

**Fig. 1:**
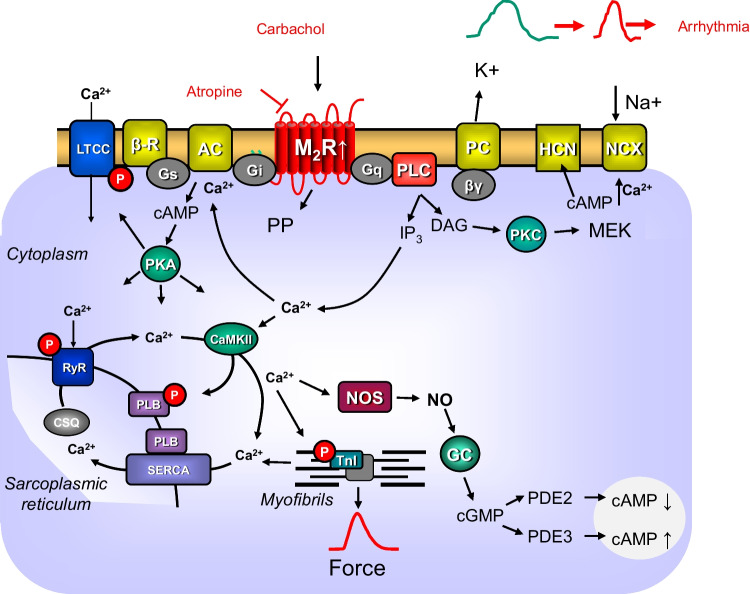
Putative mechanism of action of the M_2_-muscarinic receptor in the mammalian heart. Scheme of the signal transduction of M_2_-muscarinic receptors in cardiomyocytes. Stimulation of β-adrenoceptors (β-R) by isoprenaline leads via stimulatory GTP-binding proteins (Gs) to a production of cAMP. The increase of cAMP leads to increases in force of contraction. Carbachol stimulates M_2_-muscarinic receptors (M_2_R). In the M_2_-TG, the M_2_-muscarinic receptors are overexpressed (indicated by a white arrow pointing upwards). M_2_R are blocked by atropine. Activated M_2_R may open potassium channels (PC) via the beta and gamma subunits of inhibitory GTP-binding proteins (βγ) leading to shortening of the action potential (change from green to red symbolic action potential) culminating in an arrhythmia. Carbachol may inhibit the formation of cAMP via inhibitory GTP-binding (Gi) can reduce the activity of adenylyl cyclase (AC). Carbachol may activate protein phosphatases (PP). When carbachol reduces the level of cAMP, this may inhibit the activity of cAMP-dependent protein kinases (PKA). This leads to a reduced phosphorylation state of L-type calcium channels (LTCC). In this way, carbachol may inhibit the function of the LTCC. The hyperpolarization-activated cation channel (HCN), in sinus node cells, is opened by cAMP and this can increase the flow of cations into the cell. This depolarizes the cardiomyocytes and the beating rate in the right atrium would increases. When carbachol would reduce cAMP levels, this would reduce the activity of the HCN. Moreover, the M_2_R via GTP-binding proteins (G_q_) may activate phospholipase C (PLC). PLC leads to a formation of diacylglycerol (DAG) and inositoltrisphosphate (IP_3_). DAG can activate protein kinase C (PKC) and then MEK, another kinase. IP_3_ can increase calcium cation concentrations (Ca^2+^). When less Ca^2+^ enters the cell via the LTCC, then less Ca^2+^ is released from the stores in the sarcoplasmic reticulum. Ca^2+^ can be pumped out of the cardiomyocyte by the electrogenic sodium/calcium cation exchanger (NCX). This could lead to depolarization and arrhythmias in the right atrium. Ca^2+^ can activate the Ca^2+^ calmodulin–dependent protein kinase (CaMKII). PKA and CaMKII phosphorylate phospholamban (PLB). This leads to de-inhibition of the Ca^2+^-pump in the sarcoplasmic reticulum (SERCA). In the sarcoplasmic reticulum, Ca^2+^ binds to calsequestrin (CSQ). Ca^2+^ exits the sarcoplasmic reticulum via the ryanodine receptor (RYR). This release is enhanced by phosphorylation of RYR. The inhibitory subunit of troponin (TnI) is phosphorylated by PKA: this enhances relaxation of the muscle. Carbachol may increase the cGMP levels in the following way: Ca^2+^ may activate the nitric oxide synthase (NOS). The NOS forms NO which can activate a guanylyl cyclase (GC). This enzyme forms cGMP. This cGMP may activate the phosphodiesterase (PDE) 2 and thus more cAMP is degraded and the cell levels of cAMP may increase in this way. Alternatively, Ca may inhibit the activity of PDE3 and this would elevate cAMP levels in the cell. Finally, force is pictured in the form of the time dependence of a single contraction in red at the bottom of the scheme in red color

In isolated left atrial preparations from humans and from many other mammals (rat, mouse, guinea pig), carbachol reduces force of contraction (FOC) when given alone. However, carbachol reduces FOC in a more accentuated fashion if the force was increased by isoprenaline (Dhein et al. 2001). The negative inotropic effects of carbachol alone have been explained by an opening of potassium channels, closure of L-type calcium ion channels (LTCC), and inhibition of the activity of adenylyl cyclases or activation of protein phosphatases (Fig. 1; Neumann et al. 1993; Dhein et al. 2001). The intrinsic beating rate of the mammalian heart is reduced by carbachol alone and also in the additional presence of isoprenaline (Dhein et al. 2001). This NCE of carbachol is explained by coupling of M_2_-muscarinic receptors to inhibition of adenylyl cyclase, subsequent release of βγ subunits of G-proteins, and thus opening of potassium channels and inactivation of hyperpolarization-activated cyclic nucleotide–gated channels (HCN) and/or inactivation of LTCC (Fig. 1, Neumann et al. 1993). In the sinus node but also in other atrial cells, carbachol reduces the duration of the action potential, probably via activation of potassium channels and this leads to atrial fibrillation (Dhein et al. 2001). We hypothesized that the overexpression of human M_2_-muscarinic receptors in the cardiomyocytes of the heart may show the opposite phenotype to the ablation of the M_2_-muscarinic receptors but similar effects as the overexpression of A_1_-adenosine receptors: a more potent negative inotropic effect and an increased incidence of arrhythmias (Neumann et al. 2003, Kirchhof et al. 2003). Hence, we tested the hypothesis that in M_2_-TG, carbachol alone or after pretreatment with isoprenaline was more potent to exert a NIE and NCE than in WT. Moreover, we tested the hypothesis that M_2_-muscarinic receptors might increase the incidence of atrial arrhythmias.

## Materials and methods

### Generation of transgenic mice

Generation of M_2_-TG has not been published before. In brief, we overexpressed the complete cDNA of the human M_2_-muscarinic receptor (NCBI reference sequence: NM_001006630.2) under the control of the heart-specific mouse full-length α-myosin heavy chain promoter using methods we published repeatedly (Gergs et al. 2024). The transgenic cassette consisted of the αMHC long promoter (5464 bp), the Kozak-Human CHRM2 CDS (1407 bp), and the rabbit β-globin polyadenylation signal (rBG pA; 522 bp). To generate the transgenic mouse line, this construct was injected into fertilized oocytes of CD1 mice. For the experiments, mice of random sex being about 120 days of age were used. The experiments were approved by the local animal protection institution.

### Human samples

Human atrial samples were obtained as in previous studies (e.g., Schwarz et al. 2024). The samples were obtained during bypass open heart surgery from eight male patients and one female patient aged 55–79 suffering from severe coronary heart disease (multiple vessels affected), hypertension, and atrial fibrillation as main cardiac morbidities. Cardiac drug therapy included acetylsalicylic acid, apixaban, furosemide, metoprolol, and statins. Patients gave written informed consent.

### Contractile studies in mice

In brief, the right or left atrial preparations from the mice were isolated and mounted in organ baths as previously described (Gergs et al. 2024; Rayo Abella et al. 2024). The bathing solution of the organ baths contained in mM: 119.8 NaCI, 5.4 KCI, 1.8 CaCl_2_, 1.05 MgCl_2_, 0.42 NaH_2_PO_4_, 22.6 NaHCO_3_, 0.05 Na_2_EDTA, 0.28 ascorbic acid, and 5.05 glucose. The solution was continuously gassed with 95% O_2_ and 5% CO_2_ and maintained at 37 °C and pH 7.4 (Neumann et al. 1994). Left atrial preparations were mounted vertically in 10 mL buffer containing organ baths under isometric conditions. They were stimulated with rectangular impulses with a Grass SD 9 stimulator (Plain City, OH, USA) for a duration of 5 ms and 10% over the stimulation threshold with field stimulation using platinum electrodes. Signals were amplified via a bridge amplifier and processed using software (Labchart) from ADInstruments (Oxford, England). Spontaneously beating right atrial preparations from mice were used to study chronotropic effects. Drug application was as follows. First in LA and RA, carbachol was cumulatively applied, and then washout occurred until baseline values for beating rate or FOC were reached. Then, 1 µM isoprenaline was given. The isoprenaline increased the FOC or beating rate in LA or RA, respectively. Thereafter, carbachol was cumulatively applied. The atria and ventricles were frozen in liquid nitrogen for radioligand binding studies. Other atria and ventricles were placed into a 5% formaldehyde-containing buffer for histological studies.

### Autoradiography

Left and right atria and ventricles from mice were cryopreserved in Tissue-Tek (Sakura Europe, Alphen aan den Rijn, The Netherlands) and stored at − 20 °C or − 78 °C. The preparation of the tissue Sects. (12 µm) and the autoradiography were performed according to a reported procedure (Rayo Abella et al. 2024) with the following modifications: [^3^H]*N*-methylscopolamine (molar activity: 2.775 TBq/mmol; Novandi, Södertälje, Sweden) was used as radioligand (concentration: 1.5 nM), Leibovitz’s L15 medium (Biomol, Hamburg, Germany) supplemented with 1% bovine serum albumin (Serva, Heidelberg, Germany) was used as binding buffer, unspecific binding was determined in the presence of 1.5 µM atropine, and the incubation time was 90 min. After radioligand binding and washing, the dried slides were kept in contact with a TR 2025 E Cytiva BAS storage phosphor screen (20 × 25 cm, Fisher Scientific, Schwerte, Germany) for 29–32 days followed by the acquisition of the images.

### Histology

Cardiac preparations from the mice were fixed in buffered 4% formaldehyde and routinely embedded in paraffin. PBS was used for all washings and dilutions. Two-micrometer-thick paraffin tissue sections were deparaffinized with xylene and ethanol followed by rehydration with graded ethanol. For pathohistological analysis, tissue sections were routinely stained with hematoxylin–eosin as reported before and markers of fibrosis were analyzed by Masson/Goldner staining (e.g., Rayo Abella et al. 2024).

### Real-time RT-PCR

Samples of murine hearts and human cardiac tissue were homogenized in liquid nitrogen and total RNA was isolated by acid phenol–guanidinium thiocyanate–chloroform extraction (TRI Reagent®, Sigma-Aldrich) according to the manufacturer’s instructions and as published before (e.g., Rayo Abella et al. 2024). To confirm the quality of isolated RNA, electrophoresis in 2% agarose gel was performed. Reverse transcription was performed on intact RNA samples with the use of High-capacity cDNA Reverse Transcription Kit with RNase inhibitors (Applied Biosystems, USA). RT-PCR was performed using SYBR™ Select Master Mix (Thermo Fisher Scientific, USA) on a QuantStudio™ 3 Real-Time PCR System (Thermo Fisher Scientific, USA). To analyze the expression of exogenous M_2_-muscarinic receptor in murine ventricles, we used primers specific to human CHRM2 gene construct: forward: CTGTCACCTTTGGTACGGCT and reverse: CTTGCTGGCTCGGGATATGT; and for endogenous mouse Chrm2 gene analysis we used forward: TCCACACCCAGGTCTCCTTT and reverse: TGCCTTCTCCCTGGATCTGG primers. Primers were designed by Primer-BLAST (Ye et al., 2012). Hypoxanthine phosphoribosyltransferase 1 (Hprt1) was used as reference gene (forward primer: TTGGGCTTACCTCACTGCTT, reverse primer: ATCATCGCTAATCACGACGC). The genes with resulting C_q_ values higher than 35 (C_q_ > 35) were considered not expressed in the tissue.

### Ligand binding

The saturation binding to muscarinic receptors in the ventricles was performed using tritiated QNB ((±)-quinuclidinyl α-hydroxydiphenylacetate, L-[benzilic-4,4’-^3^H]- (^3^H-QNB, 1.18 TBq/mmol), that was purchased from Perkin-Elmer (Boston, MA, USA). The heart tissue was weighed and homogenized in a homogenizer (Ultra-Turrax® T25 basic IKA®-Werke 24,000 revolutions per minute twice for 30 s with a 30-s break). During the homogenization process, the tubes were cooled on ice and between the pulses the tissue was also kept on ice. The homogenization solution consisted of 100 mM NaCl, 10 mM EDTA, 20 mM HEPES, and the pH was adjusted to 7.4. The homogenates were transferred into Eppendorf tubes and centrifuged for 5 min at 4 °C at 1000 × g. The supernatant was transferred into empty Eppendorf tubes and centrifuged at 32,000 × g and 4 °C for 30 min. The supernatant was poured out, 600 µl of incubation buffer (100 mM NaCl, 10 mM MgCl_2_, 20 mM HEPES, pH adjusted to 7.4) was added, and the samples were homogenized. Then, another 800 µl of incubation buffer was added, followed by vortexing. This procedure gave 1400 µl of membrane preparations for radioligand saturation binding studies and the determination of the protein concentrations by the bicinchoninic acid method.

The experiments were performed similarly as reported before (Myslivecek et al. 2008). Briefly, the amount of muscarinic binding sites (B_max_) was computed by non-linear regression of the data obtained from saturation binding experiments with the binding of 65–2000 pM [^3^H]QNB (performed in duplicate). In the atria, as the amount of tissue is limiting, only one saturating concentration of ^3^H-QNB (2000 pM) was used. The *B*_max_ was calculated according to the equation *B*_max_ = B*[*R*] + *K*_D_/[*R*], where *B* is the binding measure in fmol/µg protein, [*R*] is the ligand concentration (i.e., 2000 pM), and *K*_D_ was obtained from the saturation binding experiments on ventricular membranes. Non-specific binding was determined in the presence of 5 µM atropine. Samples were incubated in a water bath at of 24 °C with constant shaking for 60 min. Then, samples were filtered through a Whatman filter using a Brandel cell harvester and the collected membranes were washed twice with ice-cooled binding buffer. The filters were allowed to dry on air overnight and were then transferred into scintillation vials. Five milliliters of Bray’s scintillation solution was added and the activity was measured with a Beckman scintillation counter as reported before (e.g., Myslivecek et al. 2008).

### Data analysis

Data shown are means ± standard error of the mean. Statistical significance was estimated using Student’s *t*-test or the analysis of variance followed by Bonferroni’s *t*-test, or chi-square tests were applied as described in the legends. A *p*-value < 0.05 was considered to be significant.

### Drugs and materials

The drugs isoprenaline hydrochloride (#I5627) and carbachol (#PHR1511) were from Sigma-Aldrich (Taufkirchen, Germany). All other chemicals were of the highest purity grade commercially available. Deionized water was used throughout the experiments. Stock solutions were prepared freshly on the day of the experiment.

## Results

**Fig. 2:**
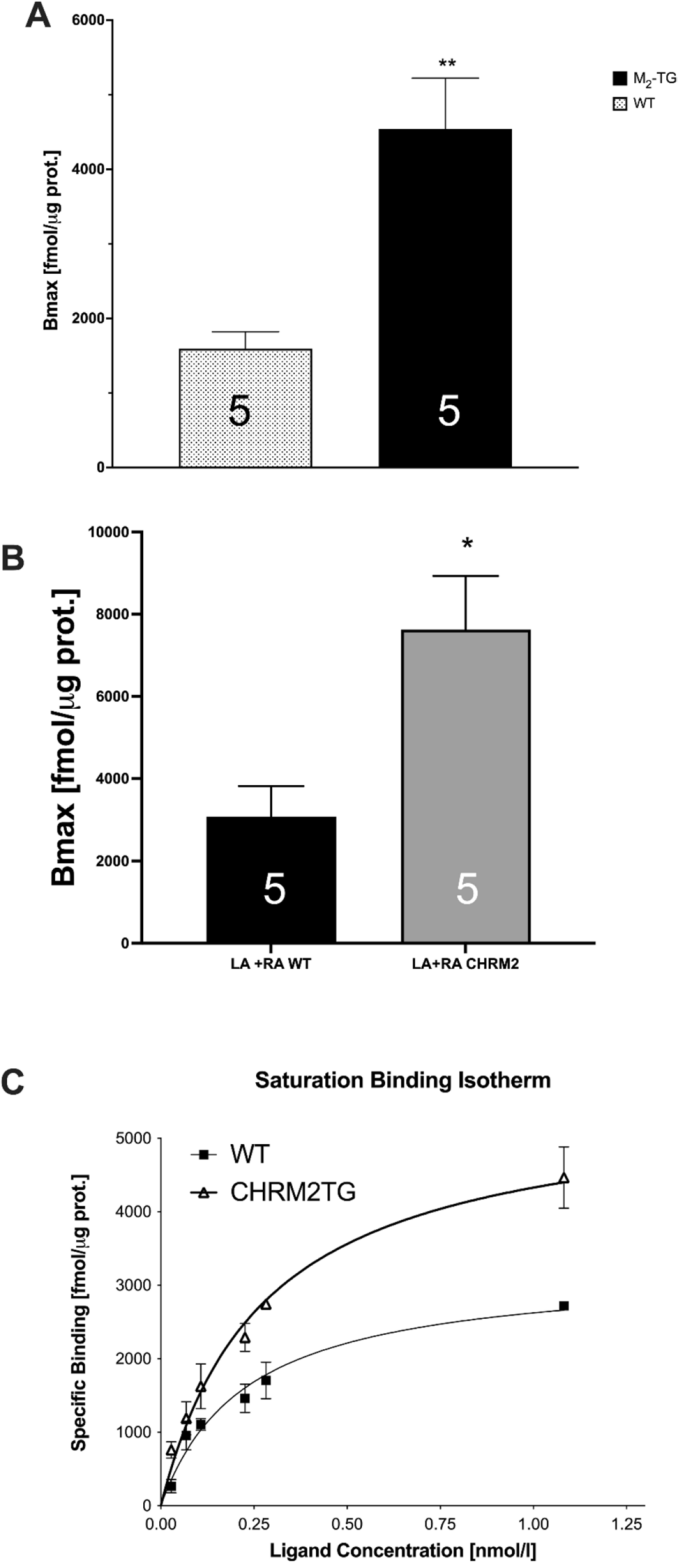
Increased protein expression of the M_2_-muscarinic receptors in M_2_-TG. **A** Saturation binding experiments with [^3^H]QNB at cardiac membranes led to the calculation of B_max_ values ± SEM (ordinate), which were higher for M_2_-TG compared to WT (**p < 0.05). Individual values are shown. Numbers in bars indicate the number of experiments. **B** Similar ligand binding results were obtained with membrane preparations from M_2_-TG atria and WT atria. The left (LA) and right (RA) atrium of each mouse had to be pooled to obtain sufficient material for binding studies. *p < 0.05 vs. WT. Numbers in bars indicate the number of experiments. **C** Saturation binding isotherm: Saturation binding experiments with [^3^H]QNB at cardiac membranes. The ordinate shows the specific binding of [^3^H]QNB at membranes of wild-type mice whole heart (WT) or the M_2_-muscarinic receptor overexpressing mice (CHRM2TG). The abscissa shows the concentration of the ligand, namely [^3^H]QNB, in nanomolar concentrations

We observed that the heart weight, body weight, and thus the relative heart weight of M_2_-TG did not differ from those of WT (data not shown). Next, we tried to characterize cardiac M_2_-muscarinic receptors in M_2_-TG. We have measured the radioligand [^3^H]QNB binding at membranes of mouse ventricles and found the increased number of muscarinic receptors in M_2_-TG mice (Fig. 2A). In more detail, we measured the equilibrium dissociation constant (K_D_) values. These K_D_ values amounted to 96 ± 30 pM in WT (*n* = 5) and were not different from those in M_2_-TG, which amounted to 82 ± 33 pM (*n* = 5, unpaired* t*-test: *p* = 0.7486).

**Fig. 3:**
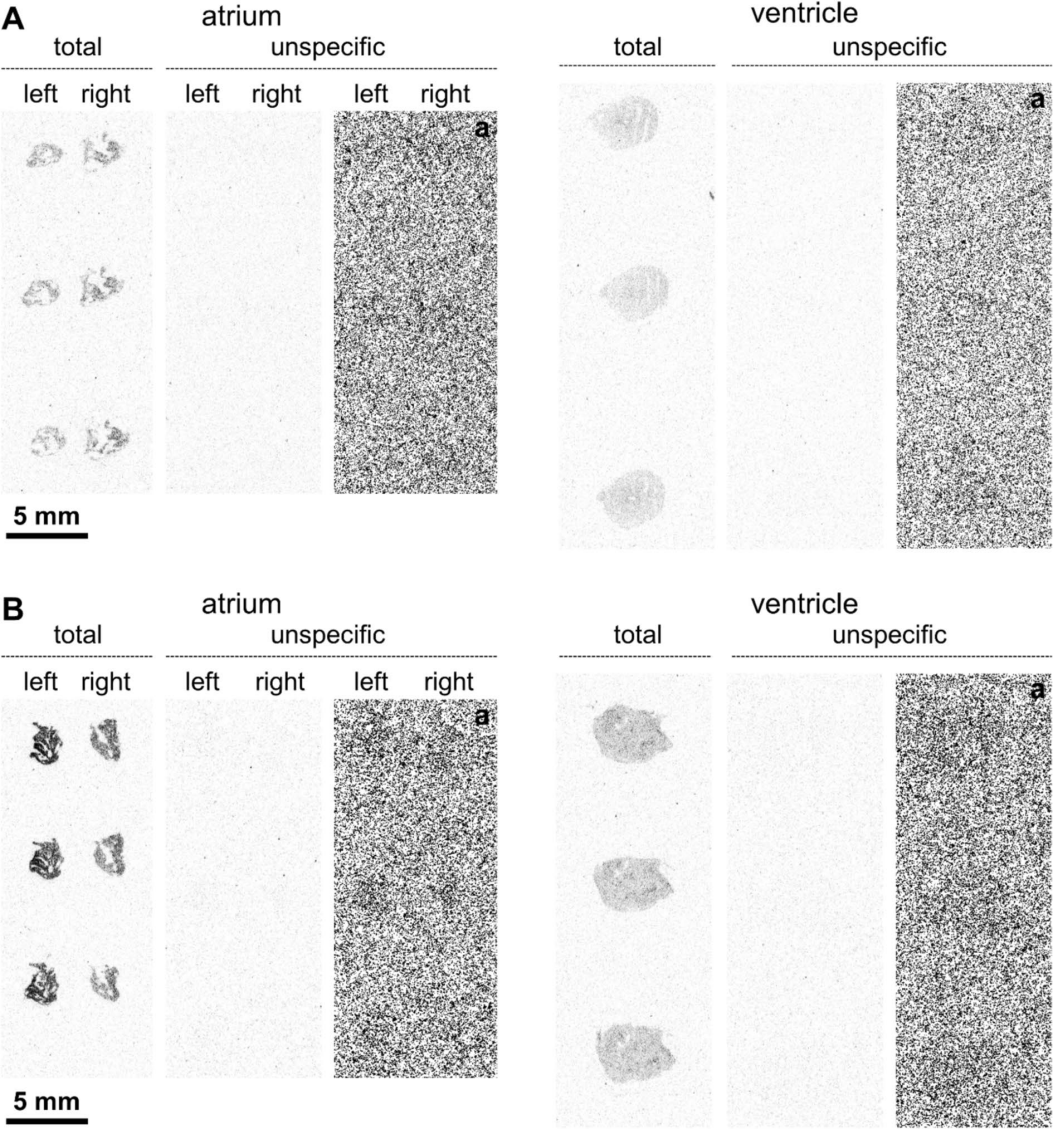
Autoradiography shows M_2_-muscarinic receptor overexpression in the atria. Detection of muscarinic receptors with [.^3^H]*N*-methylscopolamine in atrial and ventricular preparations from WT (**A**) and M_2_-TG (**B**). Shown are representative autoradiographic images from two WT and three M_2_-TG mice. The darkness of the gray shades correlates with the amount of bound radioligand. The autoradiographic images indicate that M_2_-muscarinic receptors are overexpressed in the right and left atrium of M_2_-TG compared to WT. Overexpression of M_2_ receptors in the ventricles was not evident. Note: as unspecific radioligand binding was not visible, an additional tone adjustment was applied to the images allowing visual perception of the sections (labeled as **a**)

Moreover, as we performed contraction studies in left atrial preparations (LA) and right atrial preparations (RA, vide infra), we were also interested in the expression of M_2_-muscarinic receptors in atria from WT and M_2_-TG. Due to the small amount of material, the LA and RA of each mouse had to be homogenized together. We measured that the expression of M_2_-muscarinic receptors was increased about 2.5-fold in atria from M_2_-TG compared to atria from WT (Fig. 2B). The M_2_-muscarinic receptor expression was additionally studied by autoradiography, a method we used before (Rayo Abella et al. 2024), but in the present study the radioligand [^3^H]*N*-methylscopolamine was used. This suggested higher M_2_-muscarinic receptor protein expression in M_2_-TG than in WT (Fig. 3).

**Fig. 4:**
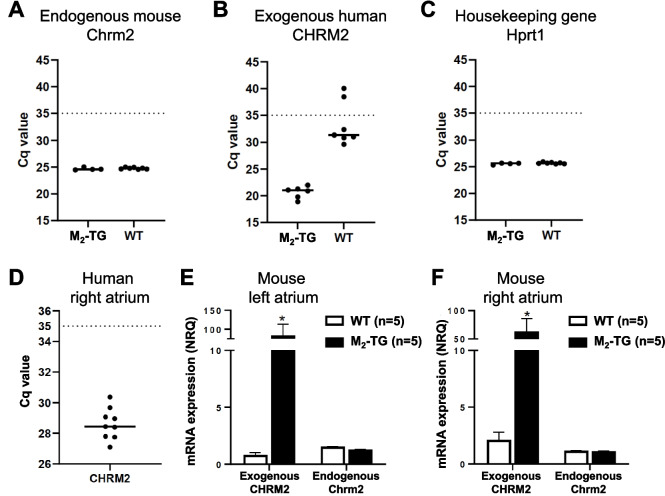
RT-PCR. Scatter plots showing the mRNA expression of the endogenous mouse M_2_-muscarinic receptor (Chrm2) (**A**), the exogenous human M_2_-muscarinic receptor (CHRM2) (**B**), and the housekeeping gene Hprt1 (**C**) in the hearts of transgenic (M_2_-TG; *n* = 4–6) and wild-type mice (WT; *n* = 7). **D** mRNA expression of CHRM2 in human atrial preparations from *n* = 9 patients. In the mouse hearts, the endogenous mouse M_2_-muscarinic receptor mRNA was found to be unchanged as was the case of a typical housekeeping gene. As a tentative approximation of the expression of the human M_2_-muscarinic receptor in human atrium, its mRNA was given. This was measured to give a possibility to assess potential clinical relevance of the model system (M_2_-TG) but direct comparison might not be possible for using different methods of quantification between mouse and man. Data are presented as C_q_ values for each measured sample. The C_q_ values higher than 35 (C_q_ > 35) are outside the set detection limit and therefore considered not expressed in the tissue. **E**, **F** Bar diagrams showing the expression of the exogenous human CHRM2 and the endogenous mouse Chrm2 in left (**E**) and right (**F**) atria of M_2_-TG (*n* = 5) and WT (*n* = 5). Data are presented as normalized relative quantity (NRQ) ± SD. *p < 0.05 vs WT (in exogenous CHRM2). Mouse Hprt1 gene was used as reference gene for normalization of gene expression

Moreover, the overexpression of the M_2_-muscarinic receptor on the messenger ribonucleotide level in the mouse heart was measured using RT-PCR (Fig. 4). More specifically, large signals for the mRNA of the human M_2_-muscarinic receptors (= exogenous receptors) were detected in the hearts of M_2_-TG compared to negligible signals in WT heart. These results are shown in Fig. 4B. The mRNA of the mouse M_2_-muscarinic receptors (= endogenous receptors) as well as the housekeeping gene Hprt1 was similarly expressed in WT and M_2_-TG and is shown in Fig. 4A and C. Amplification of the M_2_-muscarinic receptor mRNA in human atrial samples was performed using different primers with higher specificity to avoid the signals from genomic DNA (forward: ACCCAATGCCTGGCATATAGTTT, reverse: GGACTTGTAAGAGCCAGGCT) (Fig. 4D). These data suggest that atrial M_2_-muscarinic receptors were present in the human heart tissue used in the present study (Fig. 4D). Moreover, we also measured the mRNA in mouse atria. Like in the binding studies (Fig. 2B), we noticed that in the left atrium (Fig. 4E) and in the right atrium (Fig. 4F), the human M_2_-muscarinic receptor was highly expressed in the case of M_2_-TG and much less (probably missing) in atria from WT. The mRNA expression of the endogenous mouse M_2_-muscarinic receptor was similar in atria from M_2_-TG and WT (Fig. 4E and F).

**Fig. 5:**
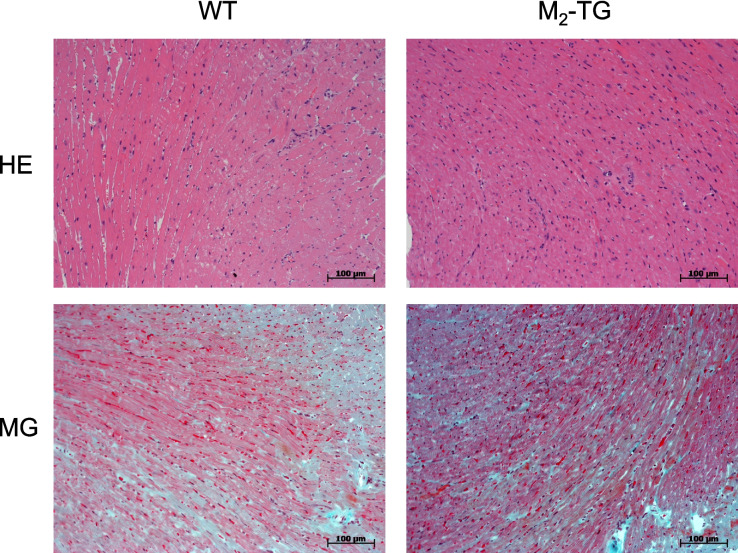
Histology. The hematoxylin–eosin (HE) staining and the Masson/Goldner (MG) staining to detect fibrosis were not different between M_2_-TG and WT hearts. The scale bars represent a distance of 100 µm

The next step was to study the histology of the M_2_-TG hearts. The HE staining did not detect changes in M_2_-TG and WT (Fig. 5). Likewise, we studied a marker of fibrosis by the Masson/Goldner staining (Fig. 5). Hence, macroscopically and microscopically, M_2_-TG does not show any disease based on the methods used here.

Finally, we wanted to study the contractile function of the overexpressed M_2_-muscarinic receptor. To this end, we performed contraction studies in the organ bath. We first applied carbachol alone in a concentration-dependent fashion. Then, we washed out carbachol and applied 1 µM isoprenaline and additionally gave cumulatively increasing concentration of carbachol. This procedure and these drug concentrations were chosen in order to detect direct and indirect (in the presence of isoprenaline) effects of carbachol on the FOC in LA and on the beating rate in RA. This protocol was used previously in our lab (Gergs et al. 2024) in different transgenic mice and was able to reveal functional differences between genotypes.

**Fig. 6:**
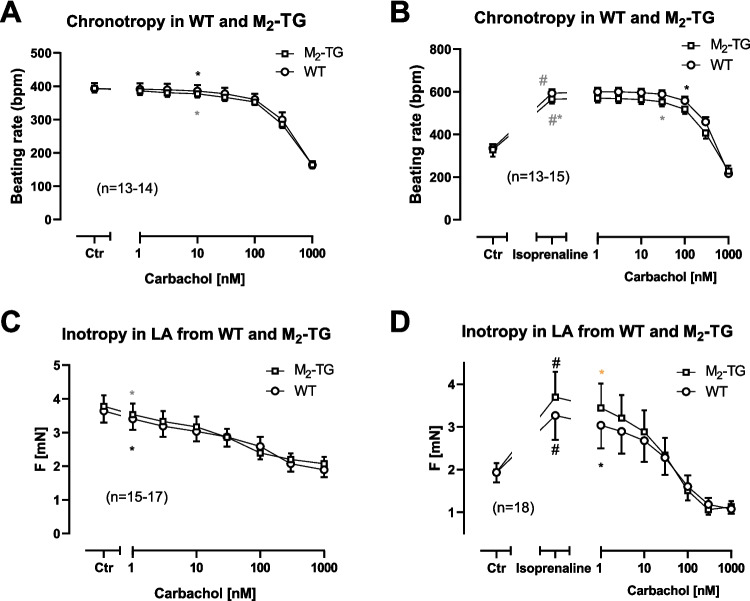
Chronotropic and inotropic effects of carbachol alone and in the presence of isoprenaline in M_2_-TG and WT atria. **A** The negative chronotropic effect of carbachol alone is similar in right atrial preparations (RA) of M_2_-TG and WT. Summarized effect of carbachol in RA from WT (circles) and M_2_-TG (squares) on beating rate in beats per minute (bpm). **B** The indirect negative chronotropic effect of carbachol is similar in RA of M_2_-TG and WT. Summarized effect of isoprenaline (1 µM) and additionally applied carbachol on beating rate in RA of WT (circles) and M_2_-TG (squares). **C** The negative inotropic effect of carbachol alone in left atrial preparations (LA) is similar in M_2_-TG (squares) and WT (circles). **D** The indirect negative inotropic effect of carbachol is similar in LA of M_2_-TG and WT. Summarized effect of isoprenaline (1 µM) and additionally applied carbachol on force of contraction in mN in LA of WT (circles) and M_2_-TG (squares). Ordinates: beating rate in beats per minute (bpm) or force of contraction millinewton (mN). Abscissae: logarithm of drug concentrations in nanomole per liter (nM). Numbers in brackets indicate number of experiments. Ctr, control values = pre-drug values. *First *p* < 0.05 vs. Ctr

In RA, the basal beating rate was the same in M_2_-TG and WT (Fig. 6A). Cumulatively applied carbachol decreased the beating rate (Fig. 6A), but this was not different between RA from M_2_-TG and WT. Isoprenaline increased the beating rate in RA from M_2_-TG and WT to the same extent (Fig. 6B). Thereafter, in the presence of isoprenaline, carbachol was cumulatively added to the organ bath. This added carbachol reduced the high beating rates that had resulted from the initial addition of isoprenaline to the organ bath. Of note, these effects were not different between genotypes (Fig. 6B).

The measurement of FOC in LA (Fig. 6C) revealed that carbachol alone reduced FOC in LA from both lines. The curves are superimposable and thus no differences in efficacy and potency of carbachol per se in the negative inotropic effect in M_2_-TG and WT exist (Fig. 6C). Thereafter, we administered isoprenaline and after 5 min, carbachol was additionally applied cumulatively (Fig. 6D). The mean value of the increased FOC was higher in M_2_-TG than in WT, but this did not reach statistical significance. Additionally applied carbachol reduced FOC without differences between LA from M_2_-TG and WT (Fig. 6D).

**Fig. 7:**
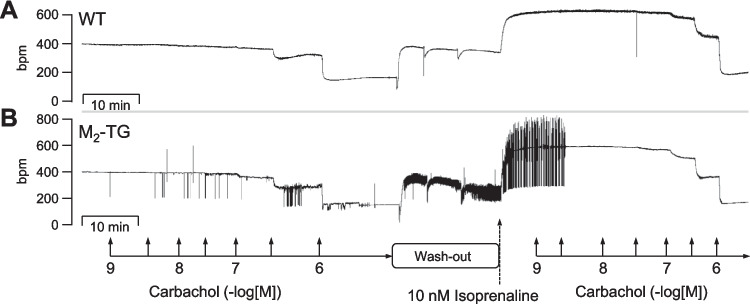
Typical manifestations of arrhythmias in right atrial preparations of M_2_-TG. The original recordings demonstrate the real-time calculation of the beating rate from the recorded force of contraction (under isometric conditions) in spontaneously beating right atrial preparations of a wild-type littermate (WT, **A**) and an M_2_-TG (**B**) on the same experimental data in adjacent organ baths. First, increasing concentrations of carbachol were cumulatively applied (10 nM to 10 µM). Then, three washout steps followed. Thereafter, isoprenaline (10 nM) was applied and in its presence, carbachol was again cumulatively applied (10 nM to 10 µM). Please note several phases of transient and finally persistent arrhythmias, which only occurred in the right atrial preparation from M_2_-TG and not from WT. Ordinates: beating rate in beats per minute (bpm). Abscissae: time in minutes (min)

Most remarkably, the incidence of arrhythmias was increased with carbachol and isoprenaline in WT and M_2_-TG. As was frequently observed in transgenic mice that we have examined for arrhythmias, we were unable to detect arrhythmias in LA of WT or M_2_-TG under the same conditions used in this study (e.g., Gergs et al. 2013; Grundig et al. 2025). However, the absolute number of arrhythmic RA under stimulation with carbachol was larger in M_2_-TG than in WT (WT: 13 or M_2_-TG: 18 from 26 each, chi-square test: *p* < 0.05). In Fig. 7, original recordings of the beating rate of a spontaneously beating RA from WT (Fig. 7A) and one RA from M_2_-TG (Fig. 7B) are presented. Here, one can visualize the multiple patterns of arrhythmias in M_2_-TG and the lack of arrhythmias in WT (Fig. 7).

## Discussion

To the best of our knowledge, this is the first description of a transgenic mouse with heart-specific overexpression of an M_2_-muscarinic receptor. Moreover, we chose to overexpress the human M_2_-muscarinic receptor to facilitate the translation of our findings into the clinic. Carbachol induced arrhythmia in living mice (Wakimoto et al. 2001; Fabritz et al. 2010) and Langendorff-perfused mouse hearts (Sassu et al. 2024). Others have described a genetic rabbit model of increased expression of the M_2_-muscarinic receptor in the heart. However, the rabbit model was obtained by crossbreeding. In this model, not only the M_2_-muscarinic receptor but also the M_3_-muscarinic receptor was highly expressed such that direct cause and effect correlations were not possible for the M_2_-muscarinic receptors. Moreover, in that model, it was not shown in which cell type of the heart the overexpression of M_2_-muscarinic took place. Furthermore, these rabbits also exhibited elevated cardiac expression of the M_3_-muscarinic receptor, which impairs a direct interpretation of their findings. Nevertheless, these rabbits show an impressive phenotype manifested in vagal overstimulation leading to prolonged RR intervals in the surface electrocardiogram of the animals. This rabbit model was developed to understand vasovagal syncope and sudden death (Livolsi et al. 2002; Chen et al. 2017). Using telemetry, such mortality studies might be possible in future studies on our M_2_-TG. In the mouse, others have achieved cardiac overexpression of the M_3_-muscarinic receptor using a similar approach as we used here, namely the use of the myosin heavy chain promoter to achieve cardiac specific overexpression of the M_3_-muscarinic receptor (Chen et al. 2017). These authors reported that M_3_-muscarinic receptor had beneficial effects on ionic currents altered in experimental cardiac hypertrophy due to aortic banding (Chen et al. 2017). Similar studies are now also planned with our M_2_-TG. M_3_-muscarinic receptors may underlie positive inotropic effects of acetylcholine at least in mice (Du et al. 1995). Stimulation of M_3_-muscarinic receptor can have antiarrhythmic effects (Liu et al. 2008). However, these receptors may lie on endothelial cells (Harada et al. 2012). Contrary to our original hypotheses, carbachol was not more effective or potent in reducing the FOC in M_2_-TG than in WT. This was true in the absence and presence of isoprenaline. We speculate here that the expression of the M_2_-muscarinic receptor is already at such a high level in the mouse heart (WT) that overexpression of the transgenic human M_2_-muscarinic receptors was unable to increase the potency of carbachol to exert negative inotropic or negative chronotropic effects in the isolated left or right atrium. Thus, for the direct effect of carbachol (given alone) or the indirect effect of carbachol (given after stimulation with isoprenaline), the additionally expressed human M_2_-muscarinic receptors are silent with regard to contractility.

However, the mRNA detection and radioligand binding studies at cardiac membranes and tissue sections clearly demonstrate increased M_2_-muscarinic receptor expression. We would argue here that conceivably the coupling of the M_2_-muscarinic receptor to effectors is too tight that overexpression cannot raise the coupling further and these overexpressed receptors are silent with respect to force and beating rate and may simply be spare receptors. They are supposedly located in different cell types, namely the sinus node cells and cardiomyocytes outside the sinus node. It would be interesting to study heterozygote KO mice for M_2_-muscarinic receptor. We would predict that a 50% reduction of M_2_-muscarinic receptor protein should attenuate the NCE and NIE mediated by M_2_-muscarinic receptors. Such experiments will be subject of future studies.

## Clinical relevance

There is in vitro evidence that carbachol via M_2_-muscarinic receptors can increase the incidence of arrhythmias in human atrial preparations (Petersen et al. 2022). In dilative cardiomyopathies autoantibodies that stimulate M_2_-muscarinic receptors have been identified that can induce deadly arrhythmias (Lazzerini et al. 2008; Nussinovitch et al. 2012; Ryabkova et al. 2019). The stimulation of M_2_-muscarinic receptors shortened the duration of the action potential and this leads to arrhythmias (Yeh et al. 2007; Dobrev et al. 2001). In ageing, the density of M_2_-muscarinic receptors declines (Swynghedauw et al. 1995; Yang et al. 2009). There is a model with M_2_-muscarinic receptor and M_3_-muscarinic receptor overexpression in the rabbit heart which may mimic SIDS (Livolsi et al. 2002, 2010a, 2010b). Moreover, our data might shed light on how overexpression of M_2_-muscarinic receptors might lead to arrhythmias in children and adult patients which has been reported by several independent research groups. Some found an increased expression of the receptor in human atrial fibrillation, some found the presence of autoantibodies that stimulate M_2_-muscarinic receptors, and others noted that stimulation of M_2_-muscarinic receptors in vitro increased the incidence of arrhythmias in human right atrial preparations (Carbajales et al. 2021; Nussinovitch et al. 2012; Ryabkova et al. 2019).

The overexpressed receptors might be spare receptors devoid of functional coupling to force or beating rate. Such spare receptors have been suggested to exist for β-adrenoceptors but also for M_2_-muscarinic receptors (Levitzki 1981; Putney and Van De Walle 1980; Dickinson et al. 1988). This hypothesis would be associated with the M_2_-muscarinic receptor-Giα-adenylyl cyclase-cyclic AMP-PKA pathway-dependent atrial muscle contraction and sinus nodal automaticity. Meanwhile, currently observed arrhythmias should occur in an alternative mechanism; for example, via the M_2_-muscarinic receptor-βγ-I_KACh_ opening pathway (Fig. 1), in which spare receptors would be absent. Regarding force of contraction, we would speculate that spare receptors exist. Hence, the pathways for force generation and the depolarization in the sinus node use different biochemical mechanisms. For instance, there is agreement that the positive inotropic effect of isoprenaline occurs via phosphorylation of LTCC, the ryanodine receptor, phospholamban, and the inhibitor subunit of troponin. Inhibition of phosphatases with phosphatase inhibitors increases the FOC and the phosphorylation state of LTCC, phospholamban, and the inhibitor subunit of troponin (Fig. 1). However, these phosphatase inhibitors failed to increase the beating rate in the mammalian heart (guinea pig right atrium: Neumann et al 1995, mouse right atrium: Schwarz et al. 2023). Hence, at least phosphatases but presumably other regulatory proteins also subserve different roles in the sinus node and in the force-generating working cardiomyocytes. Now, M_2_-muscarinic receptors may well use different signal transduction pathways, like protein phosphatases, in the sinus node of the RA compared to the force generation in the LA. In the left atrium, the negative inotropic effects of carbachol are attenuated by phosphatase inhibitors, but not the negative chronotropic effects of carbachol in the right atrium of mammals (Herzig et al 1995; Neumann and Scholz 1998; Schwarz et al. 2024).

## Limitations of the study

We have not studied human cardiomyocytes. In subsequent work, it might be possible to use stem cells to overexpress or delete M_2_-muscarinic receptors in human atrial and ventricular cardiomyocytes to directly confirm or refute our conclusions in the present model. Moreover, we did not study the ventricular function of mice. The electrophysiological mechanism(s) of arrhythmogenesis in the present M_2_-TG need(s) to be elucidated. We would predict that in M_2_-TG, carbachol would shorten the duration of the action potential more in RA from M_2_-TG than in WT, but that needs to be tested in future studies.

In summary, we achieved overexpression of human M_2_-muscarinic receptors in the heart of transgenic mice compared to the heart of WT (measured on the protein level). However, the negative inotropic and the negative chronotropic effects induced by stimulation of M_2_-muscarinic receptors in isolated left and right atria from M_2_-TG and WT were comparable. However, stimulation of mouse M_2_-muscarinic receptors induced atrial arrhythmias more often in right atrial preparations from M_2_-TG compared to right atrial preparations from WT.

## Supplementary Information

Below is the link to the electronic supplementary material.

Supplementary file1 (PDF 98 KB)

## Data Availability

All source data for this work (or generated in this study) are available upon reasonable request.
