# Supplementary figures and images for: Initial characterization of M2-muscarinic receptor overexpressing mouse heart
Source: Naunyn Schmiedebergs Arch Pharmacol. 2025 Aug 13;399(1):1467–79. doi: 10.1007/s00210-025-04502-3 (PMC12894120; doi:10.1007/s00210-025-04502-3)

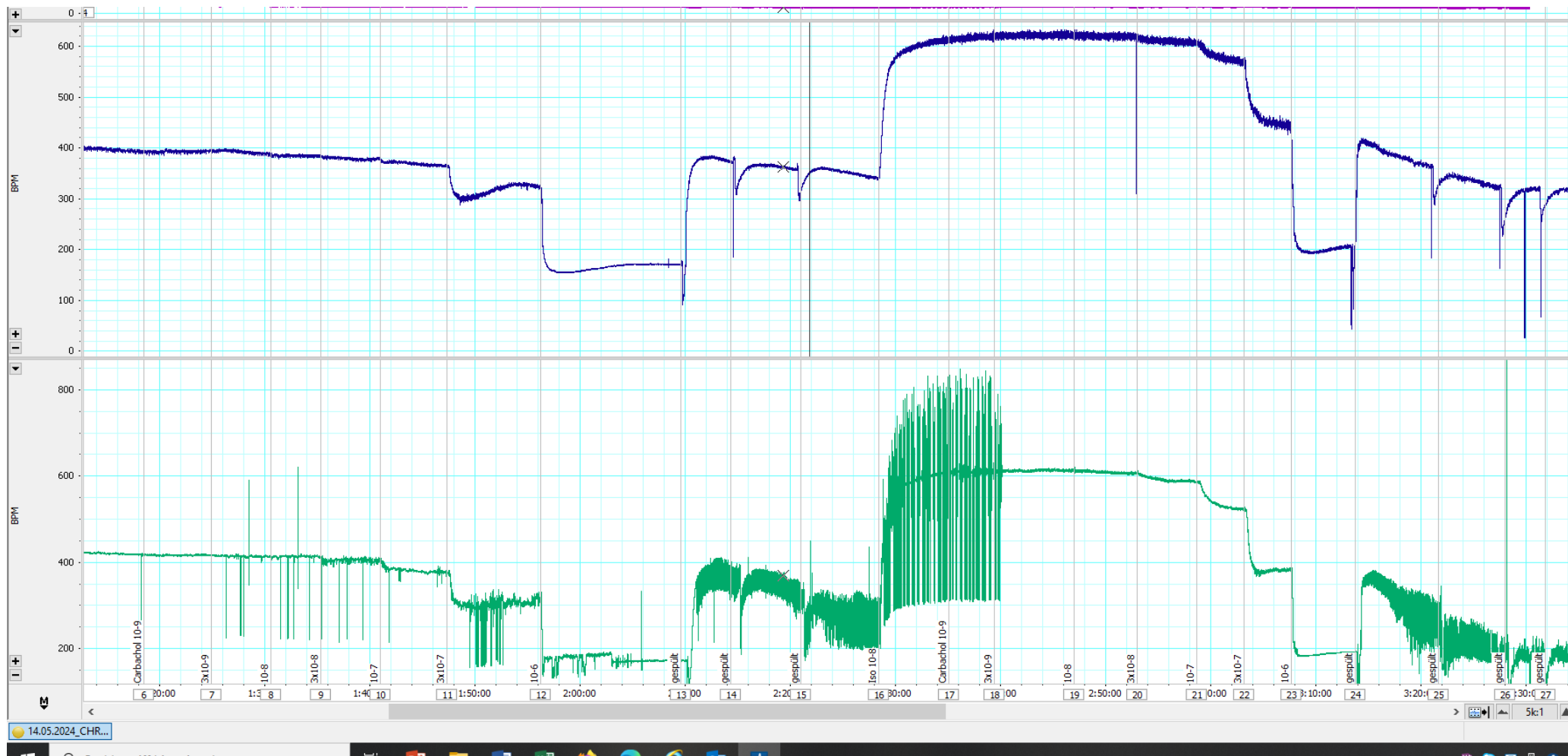

Supplement: Supplementary file 1 — Supplementary file1 (PDF 98 KB) [file 210_2025_4502_MOESM1_ESM.pdf]
